# The Culture, Community, and Science of Type 2 Diabetes Prevention in the US Associated Pacific Islands

**Published:** 2009-06-15

**Authors:** Gwen Hosey, Nia Aitaoto, Dawn Satterfield, Jane Kelly, Carter J. Apaisam, Tayna Belyeu-Camacho, Ione deBrum, Patrick Solidum Luces, Augusta Rengiil, Pasa Turituri

**Affiliations:** Centers for Disease Control and Prevention, National Center for Chronic Disease Prevention and Health Promotion, Division of Diabetes Translation. Ms Hosey is also a doctoral student at the Uniformed Services University of the Health Sciences, Bethesda, Maryland; Papa Ola Lökahi, Pacific Diabetes Education Program, Honolulu, Hawaii; Centers for Disease Control and Prevention, Atlanta, Georgia; Centers for Disease Control and Prevention, Atlanta, Georgia; DPCP Program Coordinator, Palikir, Pohnpei, Federated States of Micronesia; DPCP Coordinator, Saipan, Commonwealth of the Northern Mariana Islands; DPCP Coordinator, Majuro, Republic of the Marshall Islands; DPCP Coordinator, Hagatna, Guam; DPCP Coordinator, Koror, Palau; DPCP Coordinator, Pago Pago, American Samoa

## Abstract

**Background:**

The type 2 diabetes epidemic is a global health issue, particularly in the US Associated Pacific Islands (USAPI). Population health approaches targeting policy development and environmental transformations can help prevent or delay diabetes and related complications.

**Context:**

Since 1986, the Centers for Disease Control and Prevention, Division of Diabetes Translation has provided financial support to 6 USAPI jurisdictions for diabetes prevention and control programs. Geographic isolation, shortages of health care professionals, dependence on US and international aid, and persistent health care funding challenges are constant concerns in these jurisdictions.

**Methods:**

In September 2007, representatives from USAPI diabetes prevention and control programs, the Papa Ola Lökahi Pacific Diabetes Education Program, and the Division of Diabetes Translation met to collectively assess program goals within the Essential Public Health Services framework. Participants shared examples of integrated approaches to health promotion and diabetes prevention.

**Consequences:**

Despite persistent health care funding challenges, the assessment showed the resourcefulness of the islands' diabetes programs in leveraging resources, creating policy and environmental interventions, and strengthening connections in the traditional cultural systems.

**Interpretation:**

Population health approaches used in island jurisdictions reflect the resilience of the islands' cultures in navigating between traditional and Western ways of life. Attention to the interface of cultural knowledge and Western science provides the USAPI diabetes prevention and control programs with opportunities to create strong, sustained partnerships with the shared vision of transforming social and environmental conditions so that they can support healthy people living in healthy island communities.

## Background

The type 2 diabetes epidemic, a global health issue, is severe in the US Associated Pacific Islands (USAPI) jurisdictions. Although a post-World War II US Navy survey of the Pacific islands found no cases of diabetes, subsequent population surveys have shown a dramatic increase in diabetes (1). In 2007, the estimated prevalence of diabetes for the US population (adults aged 20 years or older, diagnosed and undiagnosed) was 10.7% (2). Although USAPI diabetes surveillance data are limited, the 2001-2003 estimated prevalence for Guam was 11% (adults aged 18 years or older, diagnosed) (3); in 2002, the estimated prevalence for Pohnpei, an island state in the Federated States of Micronesia (FSM), was 24.4% (adults aged 25-64 years, fasting blood glucose [FBG] ≥110 mg/dL) (4) and for the Republic of the Marshall Islands (RMI) was 29.8% (adults aged 15-64 years, FBG ≥110 mg/dL) (5); in 2006, the estimated prevalence for the Republic of Palau was 38.9% (all ages, FBG ≥110 mg/dL) (6); and in 2004, the estimated prevalence for American Samoa was 47.3% (adults 25-64 years, FBG ≥110 mg/dL) (7).

In the USAPI, the proportion of people with diabetes who report self-care practices to prevent complications often falls below US levels. For instance, in Guam, 2001-2003 estimated prevalence of measured hemoglobin A1c (twice annually) was 56.7% and daily self-monitoring of blood glucose (SMBG) was 32.2%, significantly lower than US estimates for measuring hemoglobin A1c (65.9%) and daily SMBG (58.3%) (3). The rates of diabetes complications are also high. For example, in 2002 and 2003, the incidence of lower limb amputations secondary to diabetes for the RMI was 79.5 per 100,000 population (8), and lower limb amputations were performed on people as young as 30 years. When standardized to the 2001 European population, the amputation incidence rate in the RMI was approximately 400 per 100,000.

Population health approaches designed to improve diabetes outcomes in health care systems and to support and sustain healthy lifestyle choices can help prevent or delay diabetes and related complications (9,10). Improving population health depends on identifying and influencing the multiple health determinants (eg, socioeconomic position, physical environment, health care access) that can influence the health of a broad population (11). Population health interventions that use policy and environmental approaches can directly affect health behaviors or influence social norms (12). These potentially low-cost, high-reach approaches can lay the groundwork for future interventions, helping to reverse the increasing prevalence of diabetes and other chronic diseases (12).

In 1986, the Centers for Disease Control and Prevention (CDC), Division of Diabetes Translation (DDT) established partnerships with the USAPI to form diabetes prevention and control programs (DPCPs). The DPCPs use funding from CDC cooperative agreements to develop expertise in diabetes prevention and control, establish systems to define the prevalence of diabetes, identify gaps in diabetes care, evaluate limited intervention projects, and develop partnerships to leverage resources and extend outreach.

In the last few years, DDT professional staff reassignments interrupted the building of collaborative relationships with the USAPI DPCPs, promulgating uncertainty related to collective diabetes prevention goals. In an effort to realign the collaborative relationships and assess shared diabetes program goals, representatives from DDT and USAPI DPCPs met in September 2007. Using the Essential Public Health Services framework (13) as a conceptual foundation, we discuss the assessment process and describe several culturally specific, community-based diabetes prevention and control strategies established by the USAPI. We also explore the importance of acknowledging the interface between Western science-based practices and indigenous knowledge as an effective population health approach in indigenous populations.

## Context

**Figure 1.**
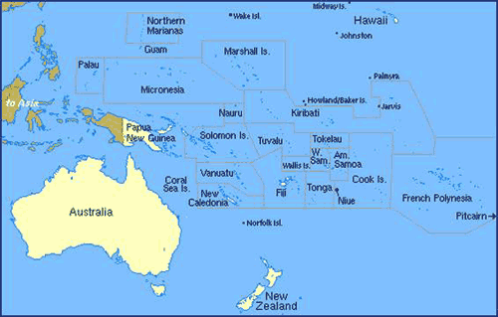
The US Associated Pacific Islands. Reproduced with permission from the Network Startup Resource Center, University of Oregon, Eugene, Oregon. http://www.nsrc.org/OCEANIA/oceania.html.

Six USAPI jurisdictions have formal relationships with the United States: the US flag territories of American Samoa and Guam; the Commonwealth of the Northern Mariana Islands (CNMI); and the FSM (includes the islands of Chuuk, Kosrae, Pohnpei, and Yap); Republic of Palau; and the RMI, which are freely associated with the United States through compact agreements (1) (Figure 1). The population centers spread across 104 inhabited islands, covering an expanse of ocean larger than the continental United States. This geographic span, dependence on US and international aid, poorly maintained and equipped health care facilities, reliance on off-island tertiary and specialty care, and shortages of health care professionals create daunting health care challenges (1).

The prevalence of diabetes in the USAPI is increasing because of the islands' rapid population and economic growth, coupled with ongoing transition from traditional ways of life (eg, communal farming and fishing) to Westernization (eg, more consumerism, with increased reliance on imported convenience foods, less physical activity) (1,14). Furthermore, a higher prevalence of obesity, 1 of the most common predisposing factors for type 2 diabetes (15), has followed on the heels of Westernization.

The USAPI annual per capita health care expenditure ranges from $140 in Chuuk to $1,032 in Guam; this stands in stark contrast to the annual US per capita expenditure of $5,711 (Table 1). Most islands depend on DDT funds to support their DPCPs. The average DDT funding award for the USAPI is $106,082, which covers administrative and staffing costs with modest support for community programs and outreach. Funding levels vary across jurisdictions depending on Congressional appropriations for the DDT competitive grant awards for DPCPs in the 50 states, territories, and island jurisdictions. Despite the persistent health care funding challenges amidst the growing epidemic of diabetes, the USAPI DPCPs have shown consistent capability and creativity in establishing partnerships across multiple sectors to leverage resources and broaden community outreach.

### Resiliency of USAPI cultures

The USAPI is a culturally diverse region composed of traditional societies with a complex integrated web of social hierarchy and traditions related to values, spiritual beliefs, and relationships with the environment. Connectivity, reciprocity, and mutual assistance are customary within strong extended family and community networks (1,14). Additionally, the USAPI share a history of colonization, resulting in challenges to their sovereignty and subsequent loss of culture, land, voice, population, health, and well-being (14,16). However, even after years of foreign occupation and influence, the USAPI cultures remain vibrant (1). To understand the approaches necessary to protect the health of the people living in island communities, one must recognize the resilience of the Pacific peoples in upholding their cultural systems while navigating between indigenous and Western lifestyles, political and spiritual ideologies, and economic systems (14,16).

### Essential Public Health Services

The Essential Public Health Services (EPHS) framework originated from the 1988 Institute of Medicine report, *The Future of the Public's Health in the 21st Century,* which outlines 3 core public health functions: 1) assessment of health status, 2) assurance of quality and accessibility of health services, and 3) policy development (17). The core functions cover the key components of public health but were difficult to define and explain to the public, policy makers, and public health professionals. In 1994, representatives from government and national public health agencies established a broad consensus definition of the EPHS (17).

**Figure 2.**
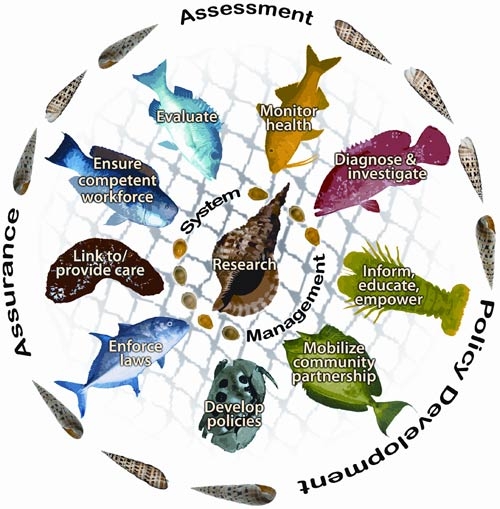
The Essential Public Health Services (EPHS) Pacific island graphic interpretation illustration. This is a representation of the EPHS developed by the US Associated Pacific Islands diabetes prevention and control programs. Overlaying the graphic is fish netting, a critical tool that helps to nourish and sustain island families and communities, symbolizing the integration and interconnectedness of each EPHS. Ocean life represents the essential services within the net and the 3 core public health functions surround the net. The central conch shell signifies research and system management, essential connectors among the EPHS.

In 2003, recognizing the rapid change in the social, cultural, environmental, technological, and global environments that influence health, the Institute of Medicine released *The Future of the Public's Health in the 21st Century.* This report suggested a broader scope for the EPHS and emphasized the need for an intersectoral public health system with links between government agencies and new partners (eg, communities, businesses, media) to transform the public's health (17). The same year, DDT began supporting the EPHS as a framework for assessing DPCPs, specifically their capacity to expand intersectoral partnerships in an effort to strengthen diabetes public health systems (13). In this framework, a public health system creates benchmarks as it identifies its work in each service area. The EPHS can provide a roadmap for engaging partners in the environmental and societal transformations needed to prevent and control diabetes. Figure 2 shows a representation of the EPHS developed by the USAPI DPCPs.

## Methods

### CDC Pacific Island DPCPs participatory meeting

Traditionally, USAPI DPCP meetings were held before large annual DDT meetings with state DPCP representatives. Breaking this pattern, DDT planned a 3-day participatory meeting in September 2007 with the 6 USAPI DPCP jurisdictions in Honolulu, Hawaii. A participatory approach is a colearning and empowering process that emphasizes the engagement of all partners as a method for research translation, systems and policy development, and community capacity building (18,19).

Because inclusion and democratic communication are key tenets of a participatory approach (18,19), we consulted with USAPI DPCP coordinators about meeting details such as date and location, possible agenda items, and suggested meeting facilitator(s). DDT and the USAPI DPCPs agreed to emphasize strengthening partnerships and co-learning during the meeting and to allow adequate time for USAPI DPCPs to share their strategies for building on local cultural strengths, knowledge, and resources to address health promotion and diabetes prevention needs. USAPI DPCPs collectively recommended the manager of the Papa Ola Lökahi Pacific Diabetes Education Program (PDEP), a champion of diabetes prevention with strong connectivity and understanding of the island cultures, as the meeting facilitator. Papa Ola Lökahi's PDEP manager accepted the invitation to facilitate and helped plan the meeting.

To strengthen links with partners who worked in diabetes prevention in the Honolulu area, the meeting's first day included the USAPI Primary Care Association, Pacific Island Health Officers Association, Micronesians United, and the Hawaii DPCP. Using the EPHS framework, the second and third days were reserved for equitable interchange and mutual understanding between DDT and USAPI jurisdictions about approaches to shared diabetes prevention goals. As the meeting progressed, an environment that fostered trust and relationship building developed, which allowed participants to unveil critical discussion topics, such as DDT staffing, future funding, and the urgent need for sustained diabetes and other chronic disease prevention efforts across island jurisdictions.

## Consequences

Several key outcomes with implications for future DDT and USAPI DPCP partnership practice emerged from this meeting. The open dialogue maintained throughout the meeting allowed DDT representatives to acknowledge the DPCPs' contributions toward a shared CDC-USAPI vision of preventing diabetes and its complications among the people of the USAPI. The information shared affirmed the USAPI DPCPs' commitment to community-based diabetes prevention focused on the strengths of each jurisdiction's cultural system. DDT representatives also gained appreciation for the USAPI DPCPs' skill in creatively leveraging scarce financial resources.

Using the EPHS framework, participants shared examples of integrated, intersectoral approaches in their jurisdictions. These approaches illustrated the creativity and resourcefulness of USAPI DPCPs in developing partnerships, applying policy and environmental interventions, and strengthening connections within the traditional cultural systems (Table 2).

These USAPI approaches also provide a link for supporting future DDT partnership practices that move beyond categorical single-sector diabetes programming to support efforts that build on strengths and assets of the USAPI partners and communities. The USAPI DPCPs cultivate partnerships with community, academic, and faith-based organizations, committed leadership (elected officials and traditional), and other public health system partners working toward sustainable community-based health promotion and disease prevention efforts. Examples of these approaches include

- In American Samoa, village *pulenuus* (leaders who serve a dual role of mayor and chief) play a crucial role. Respecting this tradition, the American Samoa DPCP has developed partnerships with these leaders across the jurisdiction to gain access to village communities for diabetes awareness and education outreach. The American Samoa Community College is helping to build and sustain diabetes community outreach through health professional training and involvement with US Department of Agriculture 4-H youth programs that provide opportunities for youth (aged 8-18 years) to learn leadership, citizenship, and life skills (eg, good nutrition and physical activity habits). Working with the college, the American Samoa Department of Health, Lyndon B. Johnson Hospital, and other community partners, the American Samoa DPCP also helps support a half-hour television program highlighting healthy nutrition and lifestyle choices. Hosted by local residents, the weekly show is popular with community members.
- In the CNMI, the Roman Catholic church plays a vital role in the lives of residents. During each of the 18 days of Lasayon Matai, the rosary prayer for the dead, the deceased's family traditionally offered a full buffet meal. In 2007, the CNMI DPCP worked with the church to adopt a policy limiting feasting to the day of the funeral. This new policy helps create a healthy, supportive environment for people living with or at risk for diabetes. The CNMI DPCP also partners with the local health care system in supporting weekly beach walks that promote physical activity and health awareness.
- To be accepted by FSM residents, diabetes prevention messages must fit into the appropriate cultural context. The FSM DPCP, respecting Chuuk's matrilineal culture, has formed a partnership with the Chuuk Women Action Council to provide diabetes outreach and management across the main island, remote lagoon islands, and outer islands. The women receive training in effective ways to promote health, tailoring their messages to fit their community's environment, language, and cultural and social customs. For example, the council's Clear the Path campaign promoted protection from foot injury by helping keep community foot paths free of debris.
- Building and sustaining links with tobacco control programs is vital to protecting the health of USAPI communities. People with diabetes who use tobacco are at greater risk for developing complications. In 2003, the median prevalence of smoking among adults (aged 18 years or older) in Guam was 38%, significantly higher than the US mainland's median prevalence of 22% for the same age group (20). The Guam DPCP works to build tobacco control and cessation partnerships within the territory of Guam, striving to reach people with diabetes while remaining sensitive to the cultural mores of connectivity, reciprocity, and mutual caregiving. In 2007, Guam's diabetes and tobacco programs collaborated to train health professionals and community members across the territory on tobacco cessation interventions in connection with the launch of Guam's tobacco quitline in September 2007. Fourteen percent of the quitline calls are from people who also report having diabetes.
- Recognizing the complexities of the Palauan language, the Palau DPCP has worked with traditional leaders to ensure accurate cultural and linguistic translation of diabetes education materials distributed at community centers, clinics, and hospitals. The Palau DPCP also supports a community group called "Di-reng" (which translates from the Pauluan as "only will power of the heart") in partnership with Belau National Hospital. Group members chose the name to show their commitment to making small, steady changes that can lead to weight loss and prevention of chronic illness such as diabetes.
- The RMI DPCP ties its diabetes outreach programs to existing cultural and community events. For example, Father's Day is one of the largest community events in the RMI; the DPCP has worked with community partners to integrate health seminars specific for men into the community Father's Day celebrations. These seminars are held separately for men, according to cultural customs, to allow men to speak freely with each other. Many of the RMI male traditional, community, and business leaders who attended these seminars now help to develop similar health promotion and diabetes prevention programs at workplaces and at other community events.

The meeting offered an opportunity for the DDT representatives, working with the meeting facilitator and USAPI DPCP participants, to realign collaborative relationships and common goals for continued USAPI capacity building. The stories shared during the meeting highlighted the success of using local cultural strength and knowledge to enhance opportunities for health promotion and diabetes prevention within USAPI communities. Recognizing the value of sharing these success stories with broader audiences that are also interested in addressing health disparities in their populations, the group presented an overview of island diabetes prevention efforts, called *Casting Our Nets for Diabetes Prevention and Control — Pulling in Our Stories,* at the DDT national conference, May 5-8, 2008, in Orlando, Florida. This was the first time the USAPI DPCPs have formally and collectively shared their stories about resourceful partnerships with CDC and other partners.

## Interpretation

The USAPI DPCPs face challenges in building capacity for diabetes prevention not shared by mainland DPCPs. Nevertheless, the USAPI DPCPs have leveraged their community connections to enhance and support policy and environmental interventions. The connecting element among indigenous cultures is a strong traditional sense of unity with the environment (16). Approaches to health issues surrounding indigenous peoples need to consider their cultural knowledge and worldview. As the links between modern health problems and separation of people from their natural environment become clearer, the interface between cultural knowledge and Western-based science, coupled with participatory approaches, may help identify broad-ranging approaches to population health. Facilitating collaborative, equitable partnerships across the interface may also be a key element in empowering communities to effectively address the social and environmental determinants of health.

The USAPI DPCPs strive to create a balance between the diverse traditions that have shaped and sustained the USAPI with Western science's evidence-based practices. This emphasis on balancing cultural knowledge and science provides the USAPI DPCPs opportunities to create strong, sustained partnerships with a shared vision: to transform social and environmental conditions to support healthy people living in healthy island communities. The Palau Ministry of Health added a service to its EPHS framework that addresses this vital concept: "Ensuring cultural integration in disease care, health protection, and health promotion" (21). The USAPI, with their development of innovative approaches to building capacity for diabetes prevention and control, may be in a unique position to contribute to the evolving knowledge and science base needed to reduce the prevalence of diabetes and other chronic diseases in populations around the world.

## Acknowledgments

We are grateful for the involvement of several Pacific island representatives during this project, including Lynn Tenorio and Father Ryan Jimenez, Commonwealth of the Northern Mariana Islands; Kipier Lippwe, Federated States of Micronesia; Roselie Zabala and Frances N. Espres, Guam; Johannes Sermai and Donny Andrike, Republic of the Marshall Islands; and Megan Fong, Henry Ichiho, and JoAnn Tsark, Papa Ola Lökahi. We also thank Mr John Dax Moreno for sharing his artistic talent in the development of the US Associated Pacific Islands Essential Public Health Services graphic illustration.

## Figures and Tables

**Table 1:** Comparison of US Associated Pacific Islands' Populations, per Capita Health Care Expenditures, and DDT Funding Awards

Location		Population^a^	Per Capita Health Expenditure, $^b^	DDT Funding Award, 2006-2008, $
**United States**		301,290,332	5,711	270,000^c^
**American Samoa**		68,200	500	58,378
**CNMI**		84,490	519	72,478
**Guam**		167,370	1,032	200,000
**FSM**	**Chuuk**	54,016	140	144,000 (total FSM award)
	**Kosrae**	7,562	440	
	**Pohnpei**	34,570	380	
	**Yap**	11,883	260	
**Palau**		20,230	850	75,139
**RMI**		55,980	302	86,301

Abbreviations: DDT, Centers for Disease Control and Prevention, Division of Diabetes Translation; CNMI, Commonwealth of the Northern Mariana Islands; FSM, Federated States of Micronesia; RMI, Republic of the Marshall Islands.

a Source for all USAPI jurisdictions except FSM: http://www.wpro.who.int/NR/rdonlyres/9655EF2C-4917-458B-B9DF-BAA306EE18CD/0/42StatisticalTables08.pdf. Source for FSM: http://www.wpro.who.int/NR/rdonlyres/CDFA5AF3-175E-4471-BBA9-97896AF6D900/0/21MicronesiaFS08.pdf. Based on data from 2007 (American Samoa, FSM, and Palau) and 2006 (CNMI, Guam, and Marshall Islands). Source for United States: http://www.census.gov/popest/states/NST-ann-est.html. Based on data from 2007.

b Source for all USAPI jurisdictions except FSM: http://www.wpro.who.int/NR/rdonlyres/9655EF2C-4917-458B-B9DF-BAA306EE18CD/0/42StatisticalTables08.pdf. Source for FSM: http://www.wpro.who.int/countries/2008/mic/national_health_priorities.htm. Based on data from 2003 (American Samoa), 2000 (CNMI and Guam), 2005 (FSM), and 2006 (Marshall Islands and Palau). Source for United States http://www.kff.org/insurance/snapshot/chcm010307oth.cfm. Based on data from 2003.

c Average CDC award to other state health departments working to build capacity for diabetes prevention and control.

**Table 2:** Examples of Essential Public Health Services Used in the US Associated Pacific Islands (USAPI) Diabetes Prevention and Control Programs

Essential Public Health Service^a^	Examples of Pacific Island DPCP Approaches
Monitor health status to identify community health problems	American Samoa, FSM, and RMI monitor health status via the WHO-STEPwise approach to chronic disease surveillance.
	Guam monitors health status by administering the CDC BRFSS every 3 years.
	Diabetes registries are developed (at various levels of operation) across the islands through the DHHS Diabetes Health Disparities Collaborative.
Diagnose and investigate health problems and health hazards in the community	Multiple challenges face USAPI DPCPs regarding monitoring diabetes and supporting the health care delivery system, for example, shortages or inadequacy of medicine and laboratory supplies, and shortages of health care professionals.
	All DPCPs are strategically engaging partners in primary and secondary prevention efforts to help decrease strain on scarce system resources.
	All DPCPs partner with their local health care systems (hospitals, clinics, and dispensaries).
	All DPCPs, in partnership with the Papa Ola Lökahi Pacific Diabetes Education Program, conducted community discussion groups to investigate diabetes problems, barriers, strengths, and community resources.

Inform, educate, and empower people about health issues	DPCPs and the Pacific Diabetes Education Program work collaboratively to increase diabetes awareness across communities.
	All DPCPs have developed or adapted national diabetes education materials to reflect local language and culture.
	DPCPs have spearheaded the following campaigns to inform the public using cultural connections: RMI's Father's Day health screening events addressed increasing men's participation in screening and diabetes education. Guam produced a diabetes management video in collaboration with local health care professionals. CNMI hosts *Cooking with Color*, a weekly television nutrition program, and recently started a Water is Cool campaign to help decrease soda pop use and an Off the TV campaign to help increase physical activity among youth.
	Palau works with traditional leaders to develop and distribute the publication *Control Your Diabetes* in Palauan.

Mobilize community partnerships to identify and solve health problems	CNMI DPCP partners with Commonwealth Diabetes Coalition, Rota, and Tinian Diabetes associations to provide nutrition counseling and blood glucose monitors to women with diabetes during pregnancy.
	FSM has developed strong links with the Development and Sustainability Agricultural Program working to develop school and community gardens to improve community access and behaviors around healthy food choices.
	Palau has developed a partnership with Ulkerreuil a Klenger, a nonprofit group supporting diabetes education and outreach through media, health fairs, schools, and religious programs.
	RMI created links with Women United Together for Marshall Islands to increase outreach efforts to outer atolls (islands).

Develop policies and plans that support individual and community health efforts	FSM DPCP working with UNICEF has created policy to promote breast-feeding.
	Palau DPCP partnered with the Department of Education to increase the number of sports offered after school and is working on a project to merge physical education and science.
	RMI DPCP facilitated the passage of a smoke-free workplace in government buildings and nutritional changes in schools and hospitals.
	DPCPs are working to integrate diabetes prevention into the CDC and WHO noncommunicable disease strategic plans within their jurisdictions.

Enforce laws and regulations that protect health and ensure safety	CNMI diabetes risk prevalence study in youth enabled a review of regulations affecting school environments and championed change.
	All DPCPs advocate for increased compliance with tobacco laws and regulations throughout the island communities.

Link people to personal health services and assure the provision of health care when otherwise unavailable	American Samoa integrates diabetes outreach with breast and cervical cancer programs.
	CNMI provides a weekly Walk on Wednesday outreach program targeted to uninsured or underinsured people living with or at risk for diabetes.
	Guam works with local clinics to promote free foot examinations and lipid screenings.
	FSM has translated diabetes resource and self-care manuals into 4 local languages.
	Palau created cultural programming that allows people with diabetes to base their behavioral choices on diabetes education networks.
	RMI created Power to the People diabetes messages to empower people with diabetes to ask for screening examinations for diabetes complications.

Assure a competent public health and personal health care workforce	Every DPCP is challenged to provide this Essential Public Health Service.
	Each DPCP attempts to work with partners (eg, hospital and dispensary systems, land grant colleges, nonprofits) to provide continuing education programs for health professionals on current evidence-based standards for diabetes care and management.

Evaluate effectiveness, accessibility, and quality of personal and population-based health services	The DHHS Diabetes Health Disparities Collaborative has been introduced within each Pacific island hospital/clinic system and is operating at various levels.
	American Samoa, FSM, and RMI are working to develop an effective interface with the WHO STEPwise approach to chronic disease surveillance.

Research for new insights and innovative solutions to health problems	American Samoa participates with Brown University in a research project on empowering community health workers to deliver diabetes education.
	CNMI conducted a cross-sectional diabetes risk prevalence study among high school sophomores, which formed the foundation for youth-oriented diabetes prevention efforts.
	FSM's Let's Go Local food campaign researched the nutritional values of locally grown fruits and vegetables and promotes local healthy food choices to the community.

Abbreviations: DPCP, diabetes prevention and control program; FSM, Federated States of Micronesia; RMI, Republic of the Marshall Islands; WHO, World Health Organization; CDC, Centers for Disease Control and Prevention; BRFSS, Behavioral Risk Factor Surveillance System; DHHS, Department of Health and Human Services; CNMI, Commonwealth of the Northern Mariana Islands; UNICEF, United Nations Children's Fund; PAHO, Pan American Health Organization.

a From the 10 Essential Public Health Services (13).
