# Erratum, Vol. 6: Issue 3

**Published:** 2010-12-15

In the article "The Culture, Community, and Science of Type 2 Diabetes Prevention in the US Associated Pacific Islands," the prevalence of type 2 diabetes in Pohnpei, Federated States of Micronesia, in 2002 was reported incorrectly. The prevalence was 32.1% (adults aged 25-64 years, fasting blood glucose [FBG] ≥126 mg/dL) instead of 24.4% (adults aged 25-64 years, FBG ≥110 mg/dL). The authors regret this error. Corrections were made to our website on September 15, 2010, and appear online at www.cdc.gov/pcd/issues/2009/jul/08_0129.htm.

